# The Electrophysiology Experience in Patients With Ebstein's Anomaly Undergoing the da Silva Cone Repair at UPMC Children's Hospital of Pittsburgh

**DOI:** 10.1111/jce.70330

**Published:** 2026-05-15

**Authors:** Brock A. Karolcik, Christopher W. Follansbee, Veenah K. Stoll, Lee B. Beerman, Jose P. da Silva, Luciana da Fonseca da Silva, Gaurav Arora

**Affiliations:** ^1^ Division of Pediatric Cardiology UPMC Children's Hospital of Pittsburgh Pittsburgh Pennsylvania USA; ^2^ Current affiliation: Baylor College of Medicine Houston TX USA; ^3^ Division of Pediatric Cardiothoracic Surgery UPMC Children's Hospital of Pittsburgh Pittsburgh Pennsylvania USA

**Keywords:** da Silva Cone repair, Ebstein's anomaly, preoperative electrophysiology evaluation

## Abstract

**Introduction:**

The da Silva Cone repair (CR) has demonstrated to be an effective intervention for Ebstein's anomaly (EA). Perioperative management can be complicated by arrhythmias, but previous studies showed improvement with preoperative catheter and intraoperative surgical ablation. The purpose of this study was to describe the evolving diagnostic protocol and perioperative findings at our center.

**Methods:**

All patients referred to UPMC Children's Hospital of Pittsburgh from February 2016 to December 2024 with EA who underwent CR were reviewed. An electrophysiology study (EPS) was performed for those with a history of Wolff–Parkinson–White, supraventricular tachycardia, ventricular tachycardia, unexplained palpitations, or syncope.

**Results:**

There were 135 patients (median age 7.8 years, range: 0.14–62 years), and 55 patients (41%) were < 5 years old at the time of CR. Seventy‐two (53%) patients had a pre‐operative arrhythmia, and 37 patients (27%) had a pre‐operative EPS at our center before CR. Nineteen (51%) had a significant finding, and 12 (32%) received a transcatheter ablation. Intraoperative ablation was performed in 86 (64%) patients with cavotricuspid isthmus (CTI) ablation performed in 74 (55%), of which 24 (18%) had a CTI with additional targeted accessory pathway ablation. Postoperative wire studies were performed in 11 (8%). Since implementing our current electrophysiologic diagnostic algorithm, 7% had a follow‐up arrythmia and only 1 underwent a postoperative EPS.

**Conclusion:**

Pre‐operative EPS, targeted surgical ablation, and follow‐up bedside wire study in EA patients with accessory pathway‐mediated arrhythmias can serve as an effective model in patients undergoing CR.

## 1 Introduction

Ebstein's anomaly (EA) of the tricuspid valve is a rare form of congenital heart disease with an estimated prevalence of 2.4 in 10,000 live births [1]. EA is characterized by apical displacement of the septal and often the posterior leaflets of the tricuspid valve due to failure of delamination of the attachments of the valves [2]. Patients with EA are prone to atrial arrhythmia, ventricular tachycardia, and ventricular pre‐excitation/Wolff–Parkinson–White (WPW) [3, 4]. WPW has been reported in 10%–30% of EA patients with > 1 accessory pathway (AP) in 20% [5, 6].

The current approach to surgical repair of the tricuspid valve in EA is the da Silva Cone repair (CR) where surgical delamination of all recruitable leaflet tissue to form a “Cone” that is anchored to the hinge point of the atrioventricular (AV) groove [7]. Our institution, through the da Silva Center for EA, serves as a major referral center for CRs.

**Figure 1:**
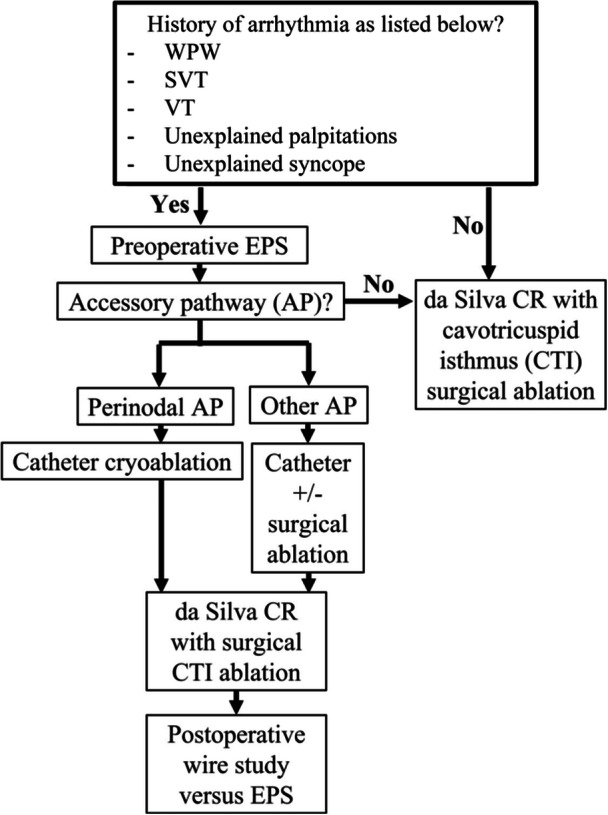
Current electrophysiology diagnostic algorithm for Ebstein's anomaly patients referred for da Silva Cone Repair at UPMC Children's Hospital of Pittsburgh. AP, accessory pathway; CR, cone repair; CTI, cavotricuspid isthmus; EA, Ebstein's anomaly; EPS, electrophysiology study; SVT, supraventricular tachycardia; VT, ventricular tachycardia; WPW, Wolff–Parkinson–White.

Over time, our institutional diagnostic protocol for electrophysiology evaluation in patients before CR has evolved. Pre‐operative EPS and intraoperative surgical ablation have previously shown improvement in postoperative EA arrhythmia burden [8, 9]. Ablation of AV APs before CR may be valuable as postoperatively these APs could be covered by valve tissue [10]. Our current institutional diagnostic protocol for all EA referral patients is to perform a pre‐operative electrophysiology study (EPS) for those with a history of WPW/ventricular pre‐excitation, supraventricular tachycardia (SVT), ventricular tachycardia, unexplained palpitations, or syncope. If an AP is identified, transcatheter ablation may be performed, depending on patient size, with additional targeted surgical AP ablation at the time of CR. Patients also receive an empiric cavotricuspid (CTI) surgical ablation at the time of CR (Figure 1). The purpose of this study was to describe the evolving diagnostic protocol and perioperative findings at our center.

## 2 Materials and Methods

A retrospective, single‐center study was conducted at UPMC Children's Hospital of Pittsburgh of all patients referred to UPMC Children's Hospital of Pittsburgh (CHP) from February 2016 to December 2024 with EA who underwent CR. This study was approved by the University of Pittsburgh Institutional Review Board and was conducted in compliance with the Health Insurance Portability and Accountability Act. The requirement for informed consent was waived due to the retrospective nature of the study. Demographics, preoperative characteristics (history of arrhythmia, use of antiarrhythmics, history of previous EPS, and preoperative EPS findings), perioperative characteristics (performance of surgical ablation, device implantation, postoperative arrhythmia, antiarrhythmic use on discharge, and postoperative wire study findings), and post‐discharge postoperative findings (time since CR, antiarrhythmic use, presence of arrhythmia, follow‐up EPS performed, EPS findings, device implantation, mortality, heart transplantation) were collected via chart review. Epic Care Everywhere (Epic Systems Corporation, Verona, Wisconsin, USA) was utilized to collect follow‐up data in domestic patients from outside institutions when available. Limited data were available for international patients. The electrophysiologic evaluation for EA patients was evaluated for the overall time period of the study (2016–2024), the pre‐standardized EA EPS diagnostic algorithm (2016–2020), and the post‐standardization of the EA EPS diagnostic algorithm (2020–2024).

For patients undergoing an EPS at our institution, since January 2021, standard diagnostic evaluation includes investigation for manifest or concealed APs and inducible SVTs. Ventricular extrastimulation protocol evaluation was performed when there was a specific concern for ventricular arrhythmia as our institutional practice was not to perform empiric ventricular extrastimulus protocols in EA that underwent EPS. Catheter ablation was attempted if a suitable substrate was identified depending on patient size. Cryotherapy was performed for perinodal APs and AVNRT substrates and radiofrequency (RF) ablation for other APs. Following EPS, findings were discussed with the surgical team to map out a strategy for surgical AP ablation if relevant, regardless of whether they were intervened upon or not. Our surgical ablation paradigm has evolved to empiric CTI ablation for all CR patients when possible and the addition of targeted AP ablation in those with a positive EPS (Figure 1). Following intracatheter CTI ablation, bidirectional block was confirmed. In small patients, surgical AP ablation was used as primary therapy to mitigate the risk of coronary injury with transcatheter RF ablation. In cases where transcatheter AP ablation was performed, surgical AP ablation was used to reinforce these ablations. Surgical ablation was performed with RF ablation or by cryoablation based on the surgeon's preference. The location of the AP was described by the electrophysiologist to the surgeons with the assistance of the three‐dimensional map, heart model, and by direct surgical visualization of the scar when ablation had been attempted. For CTI ablation, the ablation line was empirically performed with a linear ablation probe from the inferior vena cava to the proximal right ventricle in the lateral tricuspid valve region. Cryoablation was often preferred by our surgeons to minimize the risk of right coronary artery injury, especially in small patients [11].

In patients who underwent surgical AP ablation, a bedside epicardial atrial and ventricular wire study was performed to evaluate for evidence of residual AP conduction. Using a bedside temporary pacemaker and post‐surgical epicardial pacing wires, atrial and ventricular pacing are both performed to evaluate for residual AP conduction. Antegrade conduction was evaluated with incremental atrial pacing to access for AV nodal decrement and the Wenckebach cycle length without evidence of pre‐excitation. Similarly, retrograde conduction was evaluated with incremental rapid ventricular pacing to access for intermittent VA conduction with an atrial electrogram. Additionally, adenosine was given with atrial and ventricular pacing to yield the target of AV and VA block.

Operative reports were reviewed for all surgical interventions. Permanent pacemaker (PPM) and implantable cardioverter‐defibrillator (ICD) implantation related to arrhythmias were recorded. Basic descriptive statistics were performed. Categorical variables were reported as counts and percentages, while continuous variables were expressed as median and interquartile range.

## 3 Results

### 3.1 Patient Demographics

A total of 135 patients (median age 7.8 years, range: 0.14–62 years) with EA who underwent CR at our center from February 2016 to December 2024 (Table 1). Of those, 55 patients (41%) were < 5 years old at the time of CR. Sixty‐three (47%) of the patients were female. White race accounted for 101 (75%) of the patients. Thirty‐nine (29%) had public insurance. Domestic patients represented 75% of the cohort (*n* = 101). In the current era (2021–2024), 109 (80.7%) EA patients are under CR following the standardized EPS algorithm.

**Table 1:** Patient demographics of patients with Ebstein's anomaly undergoing the da Silva Cone repair at UPMC Children's Hospital of Pittsburgh.

Demographics	Era		
	Overall (*n* = 135)	2016 − 2020 (*n* = 26)	2021 − 2024 (*n* = 109)
Age at CR, total (years)	7.8 (2.3, 17.8)	9.3 (1.6, 19.8)	6.9 (2.3, 17.8)
Number of patients < 5 years old at CR	55 (40.7%)	10 (38.5%)	45 (41.3%)
Age (years), < 5 years old only	1.9 (1.1, 3.1)	1.2 (0.4, 2.4)	2.0 (1.2, 3.2)
Female sex	63 (46.7%)	16 (61.5%)	56 (51.4%)
Race			
White	109 (80.7%)	20 (76.9%)	89 (81.7%)
Black	5 (3.7%)	2 (7.7%)	3 (2.8%)
Asian	5 (3.7%)	0	5 (4.6%)
Other	16 (11.9%)	4 (15.4%)	12 (11.0%)
Ethnicity			
Non‐Hispanic/Latino	113 (83.7%)	21 (80.8%)	92 (84.4%)
Hispanic/Latino	17 (12.6%)	3 (11.5%)	14 (12.8%)
Other	5 (3.7%)	2 (7.7%)	3 (2.8%)
Country of origin			
United States of America	101 (74.8%)	25 (96.2%)	76 (69.7%)
International	34 (25.2%)	1 (3.8%)	33 (30.3%)
Insurance type			
Public	39 (28.9%)	11 (42.3%)	28 (25.7%)
Private	64 (47.4%)	14 (53.8%)	50 (45.9%)
International/self‐pay	32 (23.7%)	1 (3.8%)	31 (28.4%)

*Note:* Data are reported as number (percentage) for categorical variables or as median (25th percentile, 75th percentile) for continuous variables.

Abbreviations: CR, Cone repair; U.S., United States.

### 3.2 Preoperative Outcomes

Of the patients referred for CR (*n* = 135), 72 (53%) had a history of preoperative arrhythmia (Table 2) with SVT without baseline WPW (*n *= 28, 21%) being the most common. WPW was noted in 26 (19%) patients, of which 16 (12%) also had documented SVT. Forty‐seven (35%) were on at least one antiarrhythmic preoperatively. Fifty‐four (40%) patients had a pre‐operative EPS before CR, of which 37 (27%) were performed at our center. The majority of the EPS performed at our center were performed in the current era (2021–2024) (*n* = 33). The median age at CHP EPS was 5.2 years (range: 0.5–47.2 years) and weight was 20.7 kg (range: 7.4–109.0 kg). Twenty (54%) of the CHP EPSs had a significant finding, and 12 (32%) received a transcatheter ablation. The most common EPS finding performed at our center was a negative study (*n* = 18, 49%) followed by baseline WPW with an antegrade +/‐ retrograde AP (*n* = 14, 38%) with 11 (30%) having a single WPW (antegrade +/‐ retrograde) AP and 3 (8%) with two APs.

**Table 2:** Preoperative characteristics of patients with Ebstein's anomaly undergoing the da Silva Cone repair at UPMC Children's Hospital of Pittsburgh.

Preoperative characteristics	Era		
	Overall (*n* = 135)	2016–2020 (*n* = 26)	2021–2024 (*n* = 109)
History of preoperative arrhythmia (yes)	72 (53.3%)	15 (57.7%)	57 (52.3%)
Preoperative arrhythmia type*			
SVT (undetermined mechanism)	28	7	21
WPW with SVT	16	3	13
Atrial fibrillation	9	2	7
WPW without SVT	9	0	9
Atrial flutter	7	2	5
EAT	4	1	4
VT	3	0	3
Accelerated junctional rhythm	1	0	1
High‐grade AV block	1	0	1
Preoperative antiarrhythmic use (yes)	47 (34.8%)	11 (42.3%)	36 (56.3%)
Total number of unique preoperative antiarrhythmics	1 (1, 1)	1 (1, 1)	1 (1, 1)
History of previous OSH EPS (yes)	22 (16.3%)	5 (19.2%)	17 (15.6%)
OSH EPS findings*			
Total WPW APs	21	3	18
Negative	6	0	6
Total concealed APs	4	1	3
Atrial Flutter/IART	3	1	2
AVNRT	2	1	1
Atrial fibrillation	1	0	1
UPMC CHP EPS (yes)	37 (27.4%)	4 (66.7%)	33 (30.3%)
UPMC CHP EPS findings*			
Negative	18	0	18
Single WPW AP	11	1	10
Two Total WPW APs	3	0	3
Atrial flutter/IART	3	2	1
AVNRT	2	2	0
Concealed AP	2	1	1
Atriofascicular fiber	1	0	1
EAT	1	0	1
Sustained monomorphic VT	1	0	1
Ablation performed (yes)	12 (32.4%)	2 (50.0%)	10 (30.3%)
Ablation type			
RF	7	1	6
Cryo	5	1	4

*Note:* Data are reported as number (percentage) for categorical variables or as median (25th percentile, 75th percentile) for continuous variables.

Abbreviations: AP, accessory pathway; AV, atrioventricular; AVNRT, atrioventricular nodal reentrant tachycardia; CHP, Children's Hospital of Pittsburgh; Cryo, cryotherapy; EAT, ectopic atrial tachycardia; EPS, electrophysiology study; IART, intra‐atrial reentrant tachycardia; OSH, outside hospital; RF, radiofrequency; SVT, supraventricular tachycardia; WPW, Wolff‐Parkinson‐White; VT, ventricular tachycardia. * Some patients had more than one electrophysiology study finding.

### 3.3 Perioperative Outcomes

Intraoperative ablation was performed in 86 (64%) patients with CTI ablation performed in 74 (55%) (CTI ablation alone, *n* = 50, 37%; CTI ablation with a targeted AP ablation, *n* = 24, 18%). Table 3 demonstrates the breakdown of surgical ablations overall and by era. Eight (6%) patients required perioperative device implantation (7 PPMs, 1 ICD). Four of them with documented AV block preoperatively, and one with a history of atrial fibrillation with right bundle branch block. Only two (2%) were thought to have surgical post‐operative AV block since they both had normal sinus rhythm with no evidence of AV block pre‐operatively. One ICD was planned with CR due to preoperative ventricular tachycardia. Early postoperative arrhythmia was noted in 38 (28%) patients with VT (*n* = 15, 11%), JET (*n* = 8, 6%), and EAT (*n* = 7, 5%), most commonly observed. A postoperative wire study was performed in 11 (8%) patients with 10/11 negative and 1/11 inconclusive. Thirty‐eight (28%) of patients were discharged on at least one antiarrhythmic.

**Table 3:** Postoperative characteristics of patients with Ebstein's anomaly status post the da Silva Cone repair at UPMC Children's Hospital of Pittsburgh.

Perioperative characteristics	Era		
	Overall (*n* = 135)	2016–2020 (*n* = 26)	2021–2024 (*n* = 109)
Surgical ablation (yes)	86 (63.7%)	12 (46.2%)	74 (67.9%)
Type of surgical ablation			
CTI ablation	50	5	45
CTI ablation with focused AP ablation	24	2	22
Biatrial maze procedure	6	0	6
Focused AP ablation	5	4	1
Right atrial maze procedure	1	1	0
Perioperative device implantation (yes)	8 (5.9%)	3 (11.5%)	5 (4.6%)
Perioperative device type			
Permanent pacemaker	7	3	4
Implantable cardioverter‐defibrillator	1	0	1
Postoperative Arrhythmia (yes)	38 (28.1%)	11 (42.3%)	27 (24.8%)
Postoperative arrhythmia type^a^			
VT	15	3	12
EAT	8	1	7
JET	8	1	7
AV block	6	2	4
Atrial flutter	2	1	1
Atrial fibrillation	2	2	0
SVT (undetermined mechanism)	2	1	1
Postoperative wire study (yes)	12 (8.9%)	1 (3.8%)	11 (10.1%)
Postoperative wire study findings			
Negative	11	1	10
Indeterminate	1	0	1
Discharged on antiarrhythmic(s) (yes)	38 (28.1%)	9 (34.6%)	29 (26.6%)
Number of antiarrhythmic at discharge	1 (1, 1)	1 (1, 1)	1 (1, 1)

Abbreviations: AP, accessory pathway; AV, atrioventricular; AVNRT, atrioventricular nodal reentrant tachycardia; CR, Cone repair; CTI, cavotricuspid isthmus; EAT, ectopic atrial tachycardia; JET, junctional ectopic tachycardia; SVT, supraventricular tachycardia; WPW, Wolff‐Parkinson‐White; VT, ventricular tachycardia.

*Note:* Data are reported as number (percentage) for categorical variables or as median (25th percentile, 75th percentile) for continuous variables.

^a^ Some patients had more than one substrate.

### 3.4 Post‐Discharge Follow‐Up

Follow‐up data were available for 87 (64%) of the cohort (Table 4). The median follow‐up post‐CR was 1.6 years (range: 0.3–8.2 years). Sixteen (18%) were taking at least one antiarrhythmic, and 11 (13%) were noted to have had an arrhythmia after hospital discharge. Atrial flutter (*n* = 5, 7%) was most common post‐discharge postoperative arrhythmia. CTI‐line surgical ablation was performed in two (40%) of post‐discharge postoperative atrial flutter patients. Six (7%) of the patients had a postoperative EPS performed with atrial flutter/IART found in 2, WPW AP in 1, 2 were negative, and 1 was terminated due to clinical decompensation (complication of transhepatic access at outside institution). The patient found to have WPW with SVT had a CR performed within the first year of our program. They pre‐operatively had WPW with SVT, did not undergo an EPS, and had surgical ablations of suspected AP locations.

**Table 4:** Post‐discharge follow‐up data of patients with Ebstein's anomaly status post the da Silva Cone repair at UPMC Children's Hospital of Pittsburgh.

Follow‐up data (> 6 months post‐CR)	Era		
	Overall (*n* = 87)	2016–2020 (*n* = 29)	2021–2024 (*n* = 58)
Age at last follow‐up (years)	14.9 (7.0, 33.1)	14.4 (8.1, 22.3)	14.6 (6.6, 33.7)
Follow‐up post‐CR (years)	2.0 (1.0, 4.2)	5.5 (2.8, 7.1)	1.6 (0.7, 2.5)
Taking antiarrhythmic(s) at last follow‐up (yes)	16 (18.3%)	5 (17.2%)	11 (19.0%)
Arrhythmia at follow‐up (yes)	11 (12.6%)	6 (20.7%)	4 (6.9%)
Arrhythmia type^a^			
Atrial flutter	5	2	3
WPW without SVT	2	0	2
Atrial Fibrillation	2	1	1
SVT (undetermined mechanism)	1	1	0
WPW with SVT	1	1	0
EAT	1	0	1
VT	1	1	0
Follow‐up EPS (yes)	6 (6.9%)	5 (17.2%)	1 (1.7%)
EPS findings			
Atrial flutter/IART	2	1	1
Negative	2	2	0
WPW AP	1	1	0
Decompensated onto VA‐ECMO during transhepatic access attempt	1	1	0
Ablation performed (yes)	3	2	1
Follow‐up EPS ablation			
Cryo	2	0	0
RF	1	2	1
Follow‐up device implantation (yes)	2 (2.3%)	0	2 (3.4%)
PPM	2	—	2
Device indication			
SND	2	—	2
Deceased (yes)	2 (2.3%)	1 (3.4%)	1 (1.7%)
Orthotopic heart transplant	1 (1.1%)	1 (3.4%)	0

Abbreviations: AP, accessory pathway; CR, Cone repair; Cryo, cryotherapy; EAT, ectopic atrial tachycardia; EPS, electrophysiology study; IART, intra‐atrial reentrant tachycardia; PPM, permanent pacemaker; RF, radiofrequency; SND, sinus node dysfunction; SVT, supraventricular tachycardia; WPW, Wolff‐Parkinson‐White; VA‐ECMO, veno‐arterial extracorporeal membrane oxygenation; VT, ventricular tachycardia.

*Note:* Data are reported as number (percentage) for categorical variables or as median (25th percentile, 75th percentile) for continuous variables.

^a^ Some patients had more than one arrhythmia.

Two (2%) patients required PPM implantation for sinus node dysfunction. No ICDs were implanted in follow‐up. There were three (3%) deaths in the cohort, which were all presumed not to be arrhythmogenic in nature. One was an adult with multiple non‐cardiac comorbidities who passed from complications after an elective spinal surgery, the second was a school‐aged child who passed after a clinical decompensation during transhepatic access for an outside EPS for atrial fibrillation versus atrial flutter, and the last was a medically complex toddler who passed at home from respiratory failure secondary to a tracheostomy issue. One (1%) had undergone an orthotopic heart transplant for worsening biventricular dysfunction.

Our current institutional electrophysiologic algorithm (Figure 1) was observed in the era from January 2021 through December 2024 (*n* = 58, 43%). When adjusting for this era of our evolving electrophysiology diagnostic experience, four (7%) had a post‐discharge postoperative arrhythmia, and one (2%) required a postoperative EPS. Three (5%) had atrial flutter, one (2%) had SVT of unclear mechanism, and one (2%) had atrial fibrillation in addition to atrial flutter. Two of the atrial flutter patients underwent a surgical CTI ablation, and one received a biatrial maze procedure for a history of chronic atrial fibrillation and atrial flutter, which recurred post‐discharge. A postoperative EPS had been performed in one (2%) for atrial flutter with a finding of non‐CTI‐dependent IART.

## 4 Discussion

The purpose of this study was to describe the evolving diagnostic protocol and perioperative findings at our center in 135 EA patients referred for CR from February 2016 to December 2024. The increased risk for atrial and ventricular arrhythmias has been well documented in patients with EA [8, 9, 12, 13, 14]. For this reason, in our current algorithm, patients with suspected reentrant tachycardia undergo EPS with transcatheter ablation with subsequent surgical AP ablation for reinforcement. In patients of small size with an AP, EPS localized AP location and if not septal, surgical AP ablation was used as primary therapy (Figure 1). Following all EPS, the findings are discussed with the surgical team with mapping of potential targets for surgical ablation, where applicable. Shivapour et al. first described the diagnostic protocol for patients with EA undergoing CR at Boston Children's Hospital in response to two late postoperative sudden cardiac deaths. They found preoperative EPS to be of high diagnostic value with no further sudden deaths reported in their cohort and recommended EPS as a routine preoperative test for this population [8]. Wackel et al. then showed with a pediatric cohort of EA patients (less than 21 years old) where they identified at‐risk patients with WPW, known arrhythmia, or suspected arrhythmia for preoperative EPS, and that only 2% required ablation after CR, and that the risk for arrhythmia after CR in young patients was very low [15]. In our current electrophysiologic diagnostic era (January 2021–December 2024), post‐discharge arrhythmia burden (7%, median 1.6 years) and rate of repeat ablation (2%) were similar to those of these two major studies.

Our diagnostic protocol differs from what has been previously described in that, in addition to preoperative EPS risk stratification, we report on our center's current practice of a novel surgical ablation strategy with CTI ablation in all CR cases with targeted surgical AP ablation after preoperative EPS with a postoperative wire study in those that were found to have an AP (Figure 1). Moore et al. previously demonstrated that it was important to consider ablation before surgical intervention due to limited access to the tricuspid valve annulus after CR [10]. Surgical ablation in EA undergoing surgical repair is effective for patients with a known history of atrial fibrillation and atrial flutter [14]. Hassan et al. found in EA patients who had undergone surgical repair and concomitant Maze procedures that CTI atrial flutter was the most commonly induced arrhythmia [16]. Given the known increased risk for atrial flutter/IART in the EA population and the difficulties with catheter ablation after CR, our center routinely performs empiric CTI ablation intraoperatively to hopefully limit the possibility of future CTI‐dependent IART [3, 4, 5, 10]. Of the post‐discharge arrhythmia patients, atrial flutter/IART was present in both eras despite empiric surgical CTI ablation in the later era. One of the patients in the recent era underwent an EPS where non‐CTI‐dependent IART was reported, which would explain why empiric surgical CTI ablation would be ineffective. We are unable at this time to conclude if the CTI ablation of the other patients in the recent were ineffective since they have not undergone a post‐discharge EPS. At this time, the small sample size and lack of long‐term follow‐up limit the ability to make conclusions on differences in post‐discharge CTI‐dependent atrial flutter between eras. A longitudinal follow‐up in this population would be beneficial in a future study.

Similarly, age and size (minimum age 0.14 years, weight 7.4 kg) were not exclusive for EPS in our diagnostic protocol, which is an important consideration as CR has been successfully performed in neonates and young infants [17, 18, 19]. In these patients of smaller size that meet our indication for preoperative EPS, our diagnostic protocol is to localize the AP, and if not septal, surgical AP ablation was used as primary therapy to minimize the risk of coronary injury in small hearts with RF ablation. For septal APs, cryoablation was performed, given the proximity to the AV node and surgical ablation of the AP was not.

In patients who underwent surgical AP ablation, a bedside epicardial atrial and ventricular wire study was performed to evaluate for evidence of residual AP conduction, which, to the best of our knowledge, has not been previously described in the literature. It is felt that this can help guide follow‐up, which is often non‐invasive surveillance with electrocardiograms and Holter monitors unless there is concern for residual AP conduction that may warrant a repeat EPS.

Ventricular tachycardia and sudden death are known concern in EA patients [3, 8, 20, 21]. The incidence ranged from 0.7 per 1000 patient‐years at a single center study [22] to around 5.0 per 1000 patient‐years in a multicenter [23] and a national study [24]. In our cohort, one patient underwent ICD implantation at the time of CR for a history of VT and inducible VT during their preoperative EPS. However, it should be noted that in our institutional protocol, the ventricular extrastimulus protocol was not performed in EA patients undergoing EPS unless there was a history of VT or suspected VT. There were three mortalities, none of which were arrhythmogenic in nature as detailed in Section 3.

### 4.1 Limitations

This was a retrospective study with limitations to bias secondary to missing data and patients lost to follow‐up (no follow‐up data were available at the time of data collection for international patients). There is no true control group, and the descriptive data are too limited to evaluate the effect of our recommended EA electrophysiologic diagnostic algorithm. Medical decisions were made by the physicians taking care of the patients and varied as our algorithm evolved over the reported time of this study. Lastly, follow‐up data were limited to less than 10 years, which additional arrhythmias may increase at longer term follow‐up.

## 5 Conclusion

Pre‐operative EPS, targeted surgical AP ablation, surgical empiric CTI ablation, and follow‐up bedside wire study in EA patients with AP‐mediated arrhythmias can serve as an effective model in patients of all ages undergoing CR. Since the routine implementation of this novel electrophysiology diagnostic algorithm, the rate of arrhythmia (primarily atrial) and need for repeat EPS remains low.

## Funding

The project described was supported by the National Institutes of Health through Grant Number NHLTBI #T32HL160526.

## Conflicts of Interest

The authors declare no conflicts of interest.
